# Supplementary material for: Distributed natural gas venting offshore along the Cascadia margin
Source: Nat Commun. 2018 Aug 15;9:3264. doi: 10.1038/s41467-018-05736-x (PMC6093902; doi:10.1038/s41467-018-05736-x)
Supplement: Supplementary file 2 — Description of Additional Supplementary Files [file 41467_2018_5736_MOESM2_ESM.doc]

**Description of Additional Supplementary Files**

File Name: Supplementary Data 1

Description: Information about acoustic data utilized in this study. Listed are the cruise-ID, operating vessel, year of expedition, systems used (single-beam EK60, multibeam systems, ROV sector scanning sonar), opening beam-angle of the system used for calculating spatial coverage, frequency of acoustic signal, as well as data-source (providing agencies) and links to additional information, references, and data download sites. Abbreviations used: R/V: research vessel; CCGS: Canadian Coast Guard Ship, AUV: Autonomous underwater vehicle, ROV: remotely operated vehicle, MBARI: Monterey Bay Aquarium Research Institute, GSC: Geological Survey of Canada, ONC: Ocean Networks Canada, FSV: Fisheries Survey Vessel, E/V: Exploration Vessel.

File Name: Supplementary Data 2

Description: List of all identified gas vents across the study region of Cascadia. Included are also vent-locations based on previous publications, AUV- and ROV-observations. For abbreviations used for cruises, please see Supplementary Data 1. References are given by number as listed below data table [n.a.: not available].

File Name: Supplementary Data 3

Description: Estimated flow-rates using bubble-size distribution shown in Fig. 8a and assumptions on bubble rise rate after Leifer et al.^1^ at selected acoustic flare sites off Cascadia. Also shown are water depth and the applied depth-dependent density^2^ of methane at average seafloor temperatures using simplified depth-dependent seafloor temperature function. Note the reference ID to Supplementary Data 2. All abbreviations of cruises see Supplementary Data 1. Flow rates were defined using clean bubbles for sites in water depths < 500 m (cut-off value for the regional gas hydrate stability zone, consistent with previous work^3^), and dirty bubbles to account for gas hydrate coating for sites in water depths > 500 m.

File Name: Supplementary Data 4

Description: Comparison of average flow-rates estimated applying depth and footprint normalization, using instantaneous and tidal-integrated approach.

File Name: Supplementary Data 5

Description: Coefficients for the three-term Gaussian function (Eq. 2) of the vent forcing function over a tidal cycle.
